# Low Level Laser Therapy Reduces the Development of Lung Inflammation Induced by Formaldehyde Exposure

**DOI:** 10.1371/journal.pone.0142816

**Published:** 2015-11-16

**Authors:** Cristiane Miranda da Silva, Mayara Peres Leal, Robson Alexandre Brochetti, Tárcio Braga, Luana Beatriz Vitoretti, Niels Olsen Saraiva Câmara, Amílcar Sabino Damazo, Ana Paula Ligeiro-de-Oliveira, Maria Cristina Chavantes, Adriana Lino-dos-Santos-Franco

**Affiliations:** 1 Post Graduate Program in Biophotonics Applied to Health Sciences, University Nove de Julho (UNINOVE), São Paulo, Brazil; 2 Department of Immunology, University of São Paulo, São Paulo, Brazil; 3 Department of Basic Science in Health, Faculty of Medical Sciences, Federal University of Cuiabá, Cuiabá, Brazil; 4 Post Graduate Program in Medicine, University Nove de Julho (UNINOVE), São Paulo, Brazil; University of Rochester Medical Center, UNITED STATES

## Abstract

Lung diseases constitute an important public health problem and its growing level of concern has led to efforts for the development of new therapies, particularly for the control of lung inflammation. Low Level Laser Therapy (LLLT) has been highlighted as a non-invasive therapy with few side effects, but its mechanisms need to be better understood and explored. Considering that pollution causes several harmful effects on human health, including lung inflammation, in this study, we have used formaldehyde (FA), an environmental and occupational pollutant, for the induction of neutrophilic lung inflammation. Our objective was to investigate the local and systemic effects of LLLT after FA exposure. Male Wistar rats were exposed to FA (1%) or vehicle (distillated water) during 3 consecutive days and treated or not with LLLT (1 and 5 hours after each FA exposure). Non-manipulated rats were used as control. 24 h after the last FA exposure, we analyzed the local and systemic effects of LLLT. The treatment with LLLT reduced the development of neutrophilic lung inflammation induced by FA, as observed by the reduced number of leukocytes, mast cells degranulated, and a decreased myeloperoxidase activity in the lung. Moreover, LLLT also reduced the microvascular lung permeability in the parenchyma and the intrapulmonary bronchi. Alterations on the profile of inflammatory cytokines were evidenced by the reduced levels of IL-6 and TNF-α and the elevated levels of IL-10 in the lung. Together, our results showed that LLLT abolishes FA-induced neutrophilic lung inflammation by a reduction of the inflammatory cytokines and mast cell degranulation. This study may provide important information about the mechanisms of LLLT in lung inflammation induced by a pollutant.

## Introduction

Lung diseases are an important public health problem that affects a significant part of the population, with high social and economic costs. Thus, therapies which reduce lung inflammation and that additionally reduce the economic costs, as well as the harmful side effects, are relevant. Studies have reported the anti-inflammatory effects of Low Level Laser Therapy (LLLT) in different lung diseases, including asthma and chronic obstructive pulmonary disease [1,2,3,4,5]. The effects of LLLT on injured tissues, including proliferation, collagen synthesis, and the release of growth factors from cells, are caused by the energy delivered through light irradiation. Low level laser therapy is a non-invasive therapy with few side effects, anti-inflammatory and antioxidant actions, and backed up by a low cost. However, the mechanisms involved in LLLT need to be better explored [1,5,6,7].

Lung diseases are modulated by many factors, which are classified as predisposing, causal, and of contribution [8]. Pollutants are important causal and contributory factors that lead to serious implications for public health by showing different intensities and manifesting different latency times. Pollution causes several effects on human health, including pulmonary and systemic inflammation, behavioral and cognitive effects, changes in the caliber of the airways, vascular alterations of the heart’s rhythm, reproductive disorders, and an increased incidence of tumors, among others [9,10,11,12].

Our group has studied lung inflammation induced by exposure to formaldehyde (FA). FA is an outdoor and indoor pollutant found in many industries, including textiles, paint, resins, insulating materials, plastics, adhesives, and cosmetics [13]. In addition, FA is emitted in a domestic ambient, such as that caused by furniture, building materials, chipboards, and in heating and cooking fumes [14]. Its presence in the body is due to the absorption of exogenous sources and endogenous metabolism, since FA is a metabolite resulting from the metabolism of amino acids and xenobiotics. In earlier studies, we have shown that FA induced neutrophilic lung inflammation, modulated by mast cell degranulation, neuropeptides, an increased adhesion molecule expression, together with inflammatory cytokines, in the lung tissue [11, 15].

Considering the growth of lung diseases induced by pollutant exposure which culminate with high social and economic costs, the development of new therapies that promote low costs and principally low side effects are relevant. Thus, we decided to evaluate the effects of an LLLT that showed promisor results, by using a model of lung inflammation induced by FA, of which the mechanism is well established. For this purpose, we investigated the local and systemic effects of LLLT after an FA exposure. We quantified neutrophilic lung influxes, lung myeloperoxidase activity, lung vascular permeability, cellularity in the blood and in the bone marrow, the release and gene expression of cytokines, and of mast cell degranulation in the lung tissue. This study may provide important information about the mechanisms of LLLT with regard to lung inflammation.

## Materials and Methods

### Animals

Male 2-month-old Wistar rats were obtained from the University Nove de Julho, and maintained in a light and temperature-controlled room (12/12-hour light-dark cycle, 21 ± 2°C), with free access to food and water. The experiments were approved by the Animal Care Committee University Nove de Julho (CoEP-UNINOVE).

### Exposure to formaldehyde (FA) inhalation

The rats (5/chamber) were exposed to FA inhalation (1%, 90 min/day, 3 consecutive days). We used a standard glass chamber (20 L) coupled to an ultrasonic nebuliser device (Icel^®^, Brazil) that produces an aerosol with particles of between 0.5 and 1 micron to generate a constant airstream in an aqueous solution of formalin. It is well established that the protocol of study used induces neutrophilic lung inflammation [11,12,15,16].

### Laser therapy

Group of rats received infrared laser (CW Diode Laser- MMOptics, São Paulo, Brazil) with the following parameters: output power of 30 mW, 660 nm wavelength, 60s/point, spot size of 0.14 cm^2^, resulting in an optical power density of 210 mW cm^2^ and energy density of 12.86 J/cm^2^. The optical power was calibrated using a Newport 1835 C multi-function optical power meter (Equipland, Oklahoma Road, Sao Jose,CA, USA). The laser power was monitored during laser irradiation by collecting laser light with a partial reflection (4%) mirror. The laser irradiation dose was set at 1.8 J for 1 min [4,7,17]. The laser irradiation was performed 1 and 5h post each FA or vehicle inhalation. Nine points per application into lung region by direct contact with skin were performed. After 24 h of last FA exposure the analyses were performed.

#### Irradiation Schedule

The rats received infrared laser in nine points per application (60s/point, total 540s) into lung tract by direct contact with skin as shown above. The respiratory system was irradiated by 3 points in the trachea and 3 points in each lung lobe in order to promote irradiation of all respiratory system.

### Experimental design

The rats were divided into 3 experimental groups: B, non-manipulated rats, used to investigate the basal parameters; FA, identified as rats submitted to FA inhalation and FA+L, identified as rats subjected to FA inhalation and treated with laser. The rats were killed by sectioning the abdominal aorta under deep anesthesia with ketamine-xylazine by intraperitoneal route (100 mg/kg and 20 mg/kg, respectively) 24h after the last FA inhalation. It is important to mention that an additional group was inserted which was submitted to vehicle of FA and treated with laser. However, the results obtained in this group did not differ from B group. So, we used in all graphs only the B group.

### Evaluation of cell mobilization by quantification of cells in the blood and in the bone marrow

We utilized an automated hematology analyzer (Mindray BC 2800 Vet) by quantification of blood cells. The bone marrow cells were obtained by lavage of the femoral bone (5 ml). The fluid obtained was centrifuged (1500 rpm for 15 min at 20°C) and the supernatant was discarded, while the pellet was resuspended in 1 ml of PBS. The cells were stained with crystal violet (0.2%) and quantified in a Neubauer chamber.

### Quantification of lung neutrophilic inflammation by bronchoalveolar lavage (BAL) and myeloperoxidase activity (MPO)

Tracheostomy was performed in rats, and the bronchoalveolar space was flushed twice with phosphate-buffered saline (PBS, 20 ml total volume). The collected BAL was centrifuged (1500 rpm for 15 min at 20°C), and the resulting cell pellet was suspended in 1 ml of PBS. The cell suspensions were stained with crystal violet, and the total cell number was determined microscopically using a Neubauer chamber.

Myeloperoxidase (MPO) is considering as an index of the presence of neutrophils and was measured in the lung tissue. The lungs were perfused by pulmonary artery with PBS plus heparin (5 IU/mL). In order to normalize the MPO activity among the groups, the lung tissue was homogenized with 3 mL/g PBS containing 0.5% of hexadecyltrimethylammonium bromide and 5 ml EDTA and subsequently was centrifuged (37,000g, 15 min). Samples of tissue homogenates (10 μl) were incubated for 15 min with H_2_O_2_ and o-dianisidine. The reaction was stopped by addition of 1% NaN_3_. Absorbance was determined at 450 nm using a microplate reader (Bio-Tek Instruments, Winooski, Vt).

### Determination of lung microvascular permeability

Lung vascular permeability was assessed using the Evans blue (EB, Sigma) dye extravasation procedure. After the last FA inhalation or last LLLT administration, EB dye was injected (20 mg/Kg, iv) and the rats were killed 15 min later. The lungs were perfused via the pulmonary artery with PBS, pH 7.0 containing 5 IU/ml heparin. Subsequently, fragments of lung parenchyma, trachea and intrapulmonary bronchi were taken, weighed and incubated overnight in formamide (4 ml/g wet weight) at room temperature. The concentration of EB dye extracted to formamide was determined spectrophotometrically at 620 nm (Bio-Tek instruments) using a standard curve of EB in formamide medium (0.3–100 μg/ml). The extravasated dye was expressed as μg/g of dry tissue weight.

### Quantification of cytokines in bronchoalveolar lavage (BAL)

Cytokines levels were determined in the BAL supernatants samples. The results are expressed as pg of cytokine produced per ml. IL-6, TNF and IL-10 were quantified using ELISA kits purchased from R&D Systems (Minneapolis, MN). Determinations were made in duplicate for every sample using standard curves according to the manufacturer's specifications.

### Determination of gene expression of cytokines in the lung tissue

Lung samples were snap-frozen in liquid nitrogen. According to Invitrogen, total RNA was isolated using Trizol Reagent (Invitrogen, Carlsbad, Calif) and following the protocol RNA concentrations were determined by spectrophotometer readings at an absorbance of 260 nm. First-strand cDNAs was synthesised using the MML-V reverse transcriptase (Promega, Madison, Wisc). RT-PCR was performed using the Taqman real-time PCR assay (Applied Biosystem, USA) for the following molecules: IL-6 (Rn00561420_m1), IL-10 (Rn00563409_m1), and HPRT (Rn01527838_g1). The cycling conditions were as follows: 10 minutes at 95°C followed by 45 cycles of 20 seconds at 95°C, 20 seconds at 58°C, and 20 seconds at 72°C. Sequence Detection Software 1.9 (SDS) was used for the analysis and mRNA expression was normalised to HPRT expression.

### Lung morphological analysis

Lung fragments were fixed in 4% paraformaldehyde in 0.1 M Sorensen phosphate buffer, pH 7.4, at 4°C for 24 h, dehydrate in alcohol, clarified in xylene and embedded in parafin. Sections (5 μm thick) were prepared for morphological analysis upon toluidine blue staining on the AxioScope A1 mycroscope (Zeiss, GR). The degree of airway inflammatory cell infiltration and mast cell activation was scored in a double-blind screen. The degree of peribronchiole and perivascular inflammation was evaluated by a subjective scale of 0–4. Briefly, the scoring system for cell infiltration was 0, no cells; 1, a few cells; 2, a ring of cells 1 cell layer deep; 3, a ring of cells 2–4 cells deep; and 4, a ring of cells > 4 cells deep. Mast cells activation in the lung parenchyma and in the pleura was quantified based on a five-point system: 0, no mast cells activation; 1, <25% of activated cells; 2, 25–50% of activated cells; 3, 50–75% of activated cells; and 4, >75% of activated cells. All data were analyzed through the Software Axiovision v.3.1 (Zeiss, GR).

### Statistical analysis

The Statistical analysis was performed using the GraphPad Prism software (GraphPad Software, Inc). The normality test was performed using Kolmogorov-Smirnov test. Since the data were parametric we used one-way ANOVA followed by Student´s Newman-Keuls. Differences were considered significant when p<0.05.

## Results

### Effects of treatment with LLLT in the cellular mobilization from bone marrow and blood after FA exposure

**Fig 1:**
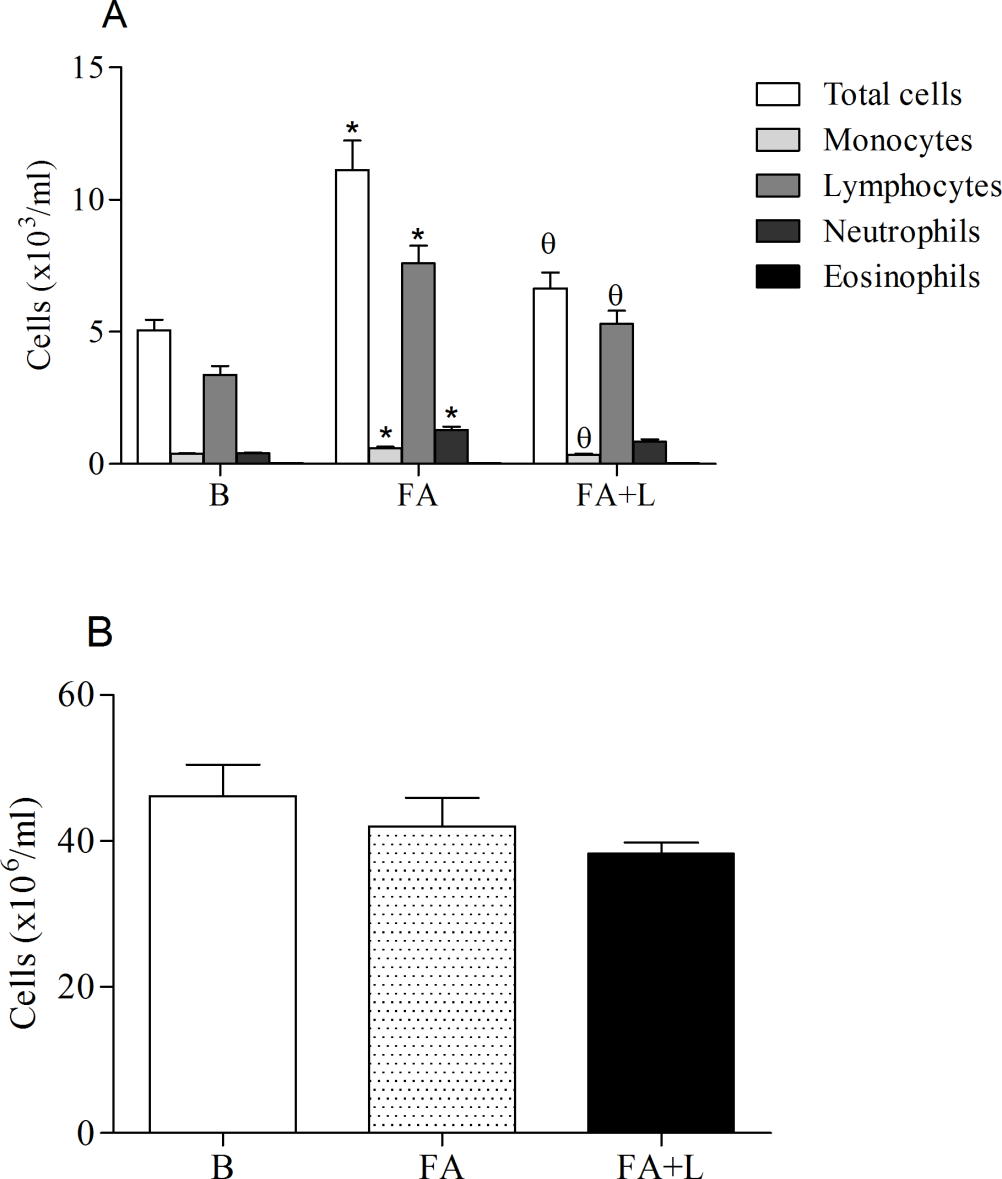
Treatment with LLLT reduces cell recruitment in the blood (A) but did not alter the number of cells in the bone marrow after FA exposure. Group of rats was exposed or not to FA inhalation (1%, 90 min/day, 3 days) and treated or not with LLLT (30mW, 1,8 J, 60s/point, total 540s, 1 and 5h post each FA inhalation). Non-manipulated rats were used to obtain basal parameters. The number of cells in the blood (panel A) and in the bone marrow (Panel B) was determined 24 h after the last FA inhalation. Data mean ± SD of 6 animals per group. *P<0.05 in relation to B group; ^θ^P<0.05 in relation to FA group.

To investigate the effects of laser therapy on cellular mobilization process, we quantified the number of cells found into the blood and into the bone marrow. Our data showed that treatment with LLLT in group of rats exposed to FA reduced the number of total cells, monocytes and lymphocytes into the blood as compared to the non-treated group (FA group). We can also observe that FA exposure increased the total cells, monocytes, lymphocytes and neutrophils into the blood in relation to rats non-submitted to FA (B group) (Fig 1, Panel A).

In panel B, we showed that there were no differences in the total cells in the bone marrow between groups of study.

### Effects of treatment with LLLT in the neutrophilic lung inflammation induced by FA exposure

To evaluate the impact of laser therapy in the neutrophilic lung inflammation induced by FA inhalation, we quantified the number of cells recovered in BAL as well as myeloperoxidase activity (MPO), which is an indirect indicator of neutrophil influx.

**Fig 2:**
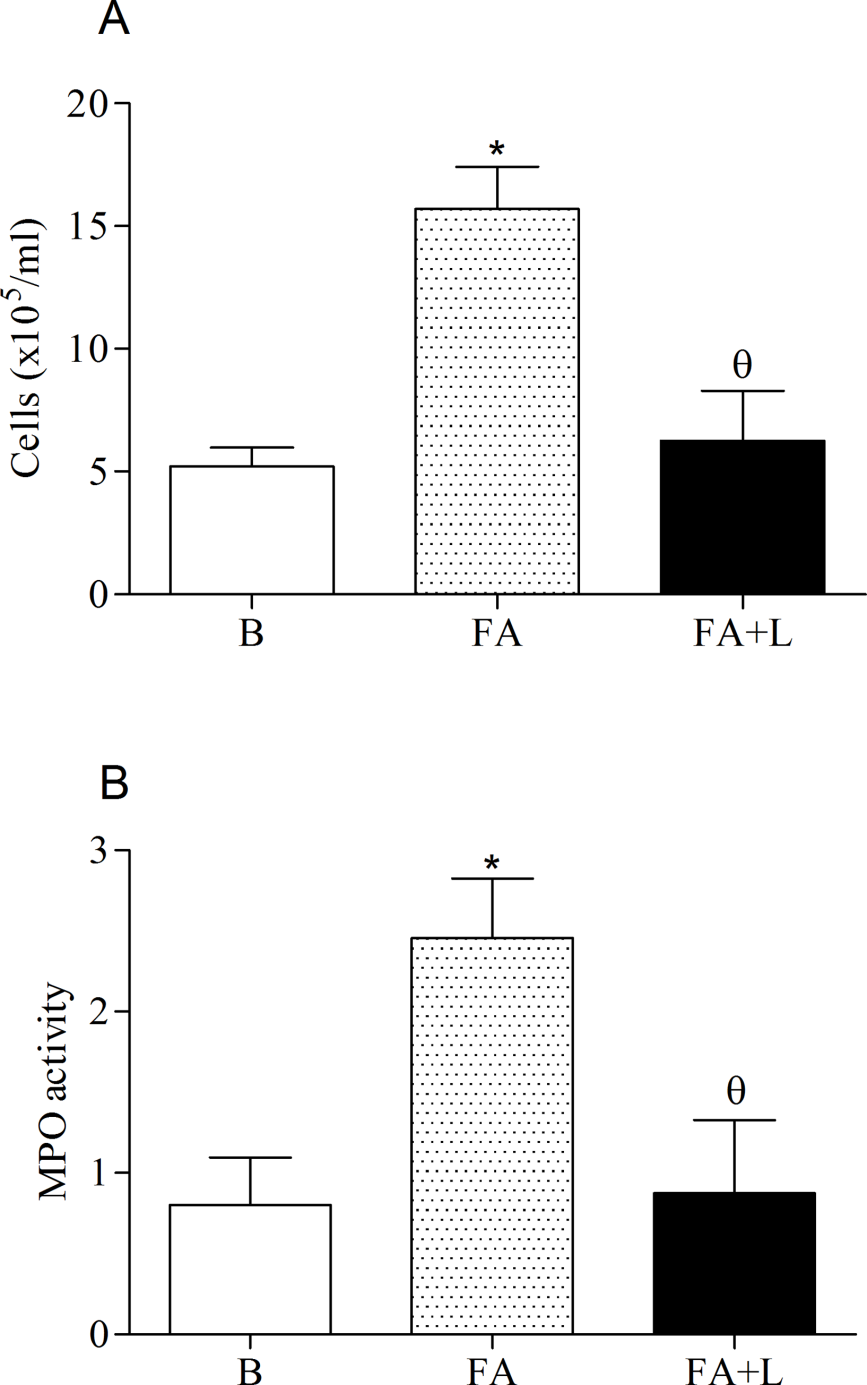
Treatment with LLLT reduces neutrophilic lung inflammation after FA exposure. Group of rats was exposed or not to FA inhalation (1%, 90 min/day, 3 days) and treated or not with LLLT (30mW, 1.8 J, 60s/point, total 540s, 1 and 5h post each FA inhalation). Non-manipulated rats were used to obtain basal parameters. The number of cells in the bronchoalveolar lavage (BAL) (panel A) and myeloperoxidase activity in the lung tissue (MPO) (Panel B) were determined 24 h after the last FA inhalation. Data mean ± SD of 6 animals per group. *P<0.05 in relation to B group; ^θ^P<0.05 in relation to FA group.

Data obtained showed that laser therapy caused a marked reduction of leukocytes into the BAL (FA+L group) in relation non-treated rats (FA group). We also confirmed increased recruitment of leukocytes into the BAL of non-treated rats (FA group) when compared to non-manipulated rats (B group) (Fig 2A).

Moreover, the effects of laser therapy on neutrophils influx were confirmed by MPO activity. Panel B showed decreased MPO activity in rats exposed to FA and treated with laser therapy (FA+L group) in relation to non-treated rats (FA group). On the other hand, FA group showed increased MPO activity in relation to B group.

### Effects of treatment with LLLT in the lung microvascular permeability after FA exposure

To evaluate the effects of laser therapy in the lung microvascular permeability, we quantified the Evans Blue (EB) extravased in the parenchyma, trachea and intrapulmonary bronchi.

**Fig 3:**
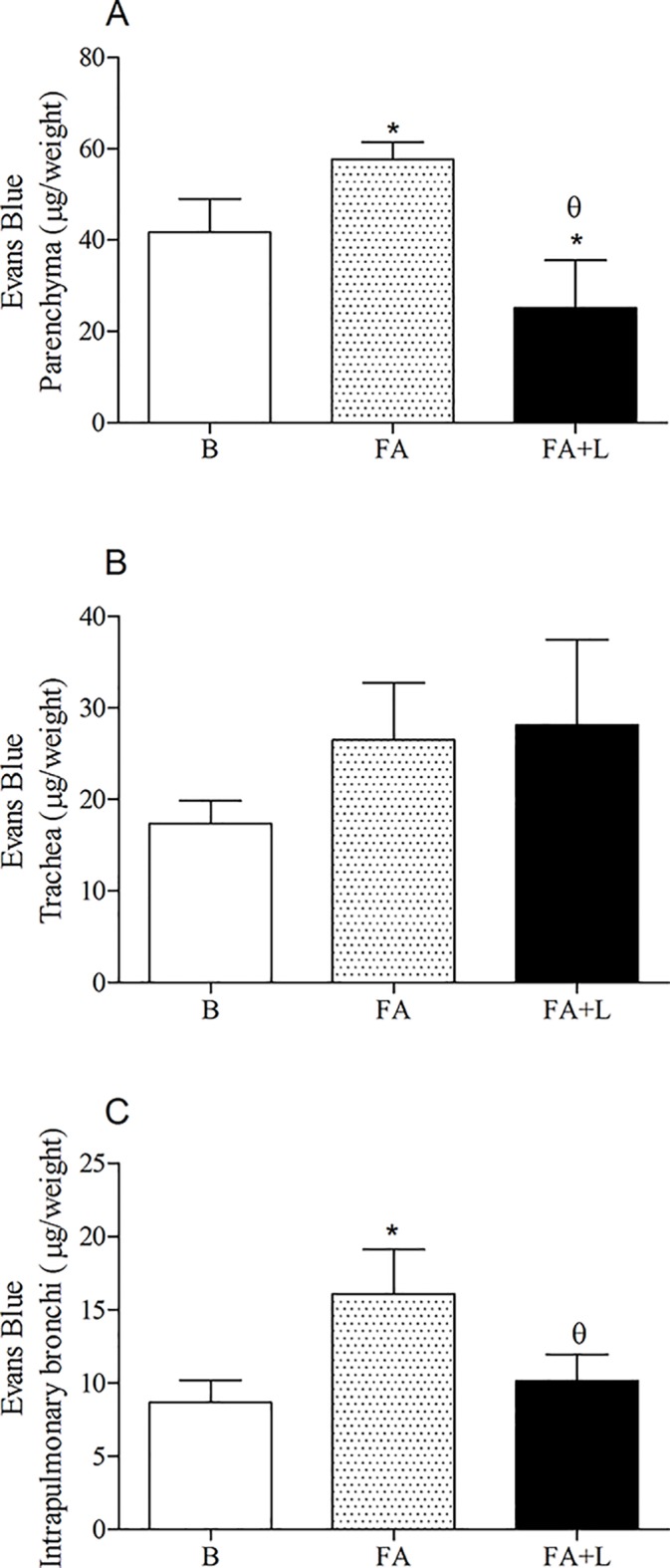
Treatment with LLLT reduces lung vascular permeability after FA exposure. Group of rats was exposed or not to FA inhalation (1%, 90 min/day, 3 days) and treated or not with LLLT (30mW, 1.8 J, 60s/point, total 540s, 1 and 5h post each FA inhalation). Non-manipulated rats were used to obtain basal parameters. The lung vascular permeability in the parenchyma (A), trachea (B) and intrapulmonary bronchi (C) was assessed immediately after the last FA inhalation. Data are mean ± SD of 6 animals per group. *P<0.05 in relation to B group; ^θ^P<0.05 in relation to FA group.

Our data of Fig 3 showed a significant reduction of Evans blue dye extravasation in the pulmonary parenchyma (Panel A) as well as intrapulmonary bronchi (Panel B) of rats treated with LLLT in relation to group of rats only exposed to FA inhalation. On the other hand, no differences were observed in Evans blue extravasation in trachea of groups of study (Panel C).

### Effects of treatment with LLLT in the cytokines released in BAL fluid after FA exposure

To evaluate the effects of laser therapy on lung inflammation induced by FA exposure, we quantified inflammatory (IL-6 and TNF) and anti-inflammatory cytokines (IL-10) in the supernatant of BAL fluid.

**Fig 4:**
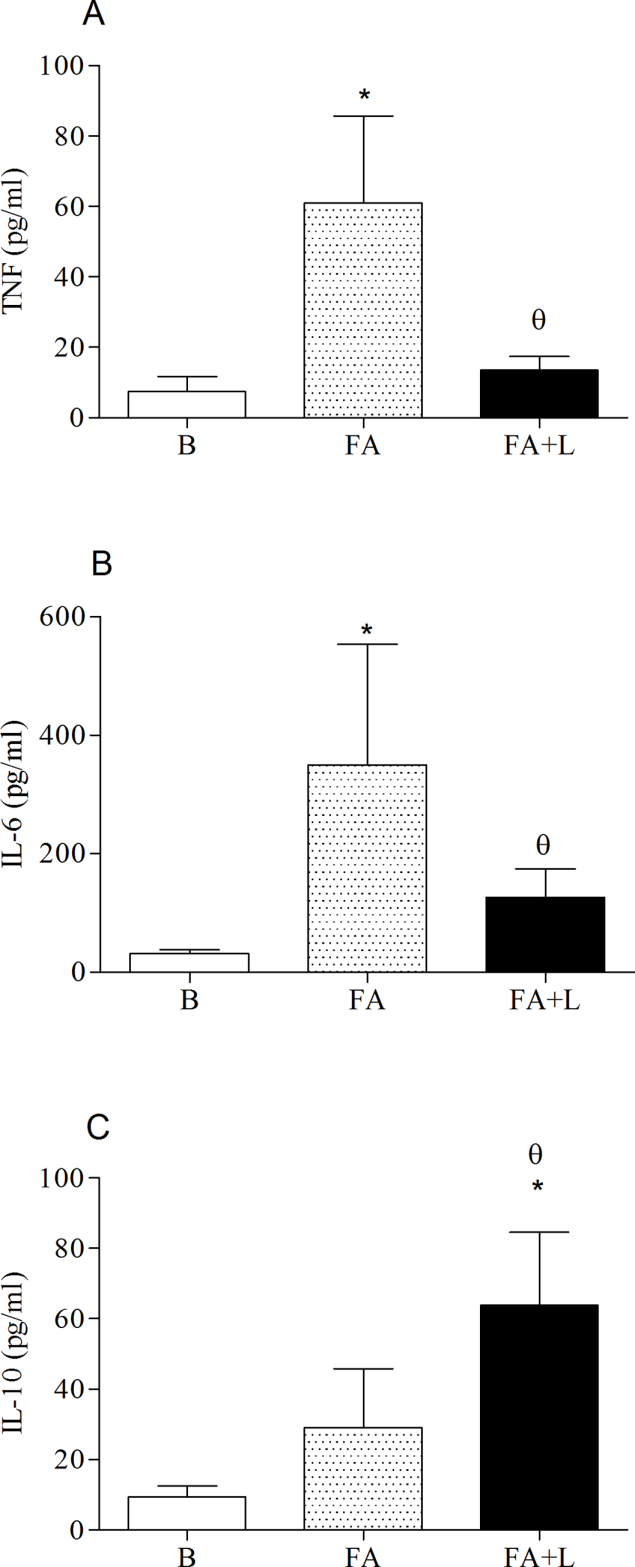
Treatment with LLLT reduces TNF-alpha and IL-6 while increases IL-10 levels in the BAL fluid after FA exposure. Group of rats was exposed or not to FA inhalation (1%, 90 min/day, 3 days) and treated or not with LLLT (30mW, 1.8 J, 60s/point, total 540s, 1 and 5h post each FA inhalation). Non-manipulated rats were used to obtain basal parameters. The evaluation of inflammatory cytokines TNF-alpha (A) and IL-6 (B) as well as anti-inflammatory IL-10 (C) in the supernatant of BAL fluid were determined 24 h after the last FA exposure. Data are mean ± SD of 6 animals per group. *P<0.05 in relation to B group; ^θ^P<0.05 in relation to FA group.

Fig 4 (Panels A and B) showed that treatment with LLLT reduced the IL-6 and TNF levels released in the BAL fluid of rats submitted to FA exposure (FA+L group) when compared to the non-treated animals (FA group). On the other hand, we can also observe that the treatment with LLLT increased IL-10 levels in BAL fluid of FA+L group in relation non-treated group (FA group) (Panel C).

In Fig 4 (Panels A, B and C) we also confirmed that FA exposure increased IL-6, TNF and IL-10 levels in supernatant of BAL fluid when compared to the B group.

### Effects of treatment with LLLT in the cytokines gene expression in the lung after FA exposure

**Fig 5:**
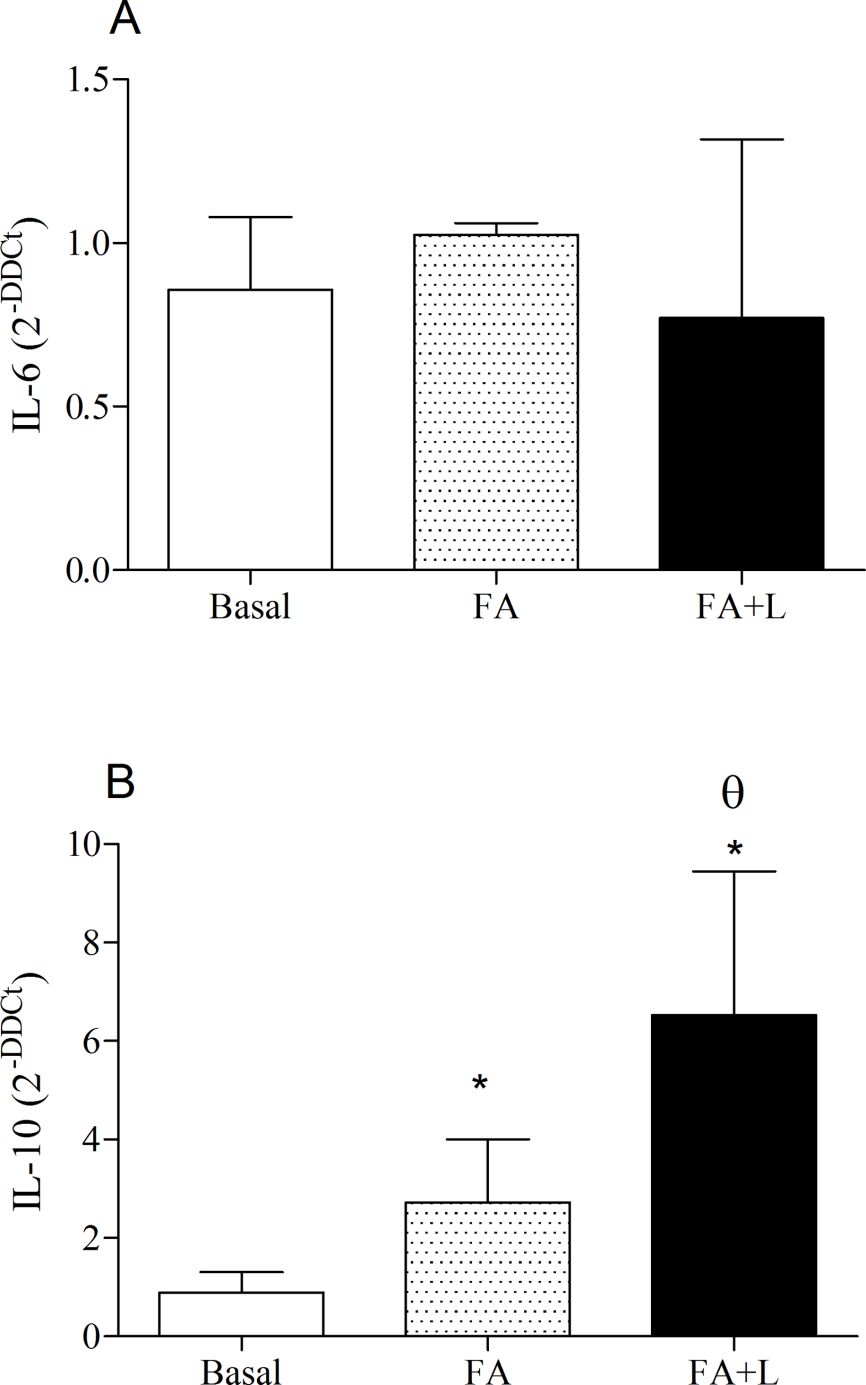
Treatment with LLLT increases IL-10 gene expression and did not alter IL-6 gene expression in the lung tissue after FA exposure. Group of rats was exposed or not to FA inhalation (1%, 90 min/day, 3 days) and treated or not with LLLT (30mW, 1.8 J, 60s/point, total 540s, 1 and 5h post each FA inhalation). Non-manipulated rats were used to obtain basal parameters. The evaluation of gene expression of inflammatory cytokine IL-6 (A) and anti-inflammatory IL-10 (B) in the lung were determined 24 h after the last FA exposure. Data are mean ± SD of 6 animals per group. *P<0.05 in relation to B group; ^θ^P<0.05 in relation to FA group (Panel B).

Fig 5 (Panel A) showed that no differences were found in the gene expression of IL-6 between groups of study. On the other hand, we can observe in panel B that the treatment with LLLT increased gene expression of IL-10 in the lung tissue in relation non-treated group (FA group).

### Effects of treatment with LLLT in the leukocytes infiltration and mast cells activation

**Fig 6:**
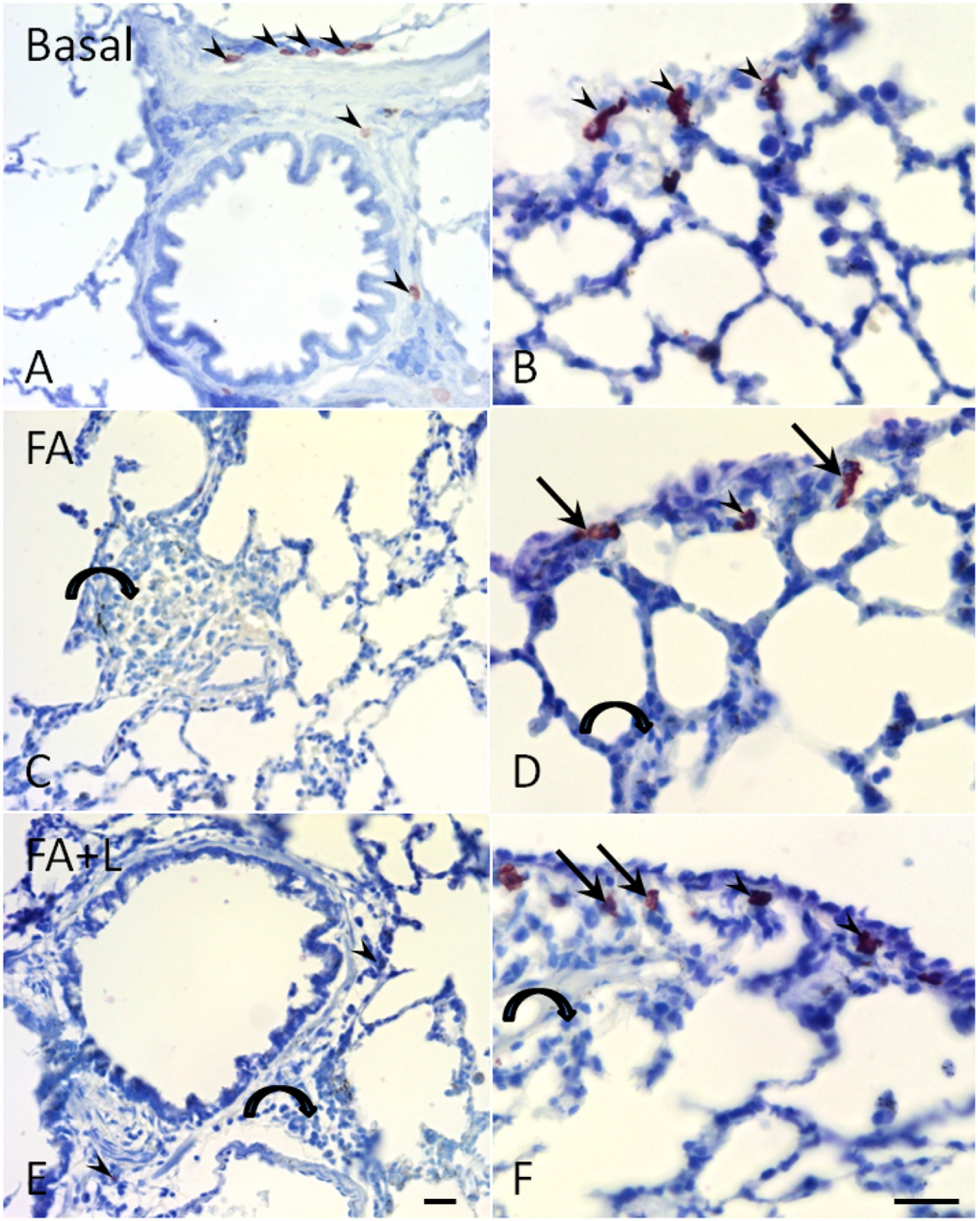
Treatment with LLLT reduces leukocytes infiltration and mast cell degranulation in the lung tissue after FA exposure. The airway inflammation induced by FA inhalation caused an intense cellular infiltration in the perivascular and peribrochiolar and mast cell activation in the lung parenchyma and pleura (arrows) (Panels C and D), whereas in the basal group, only resident leukocytes were observed and the mast cells were intact (arrowhead) (Panels A and B). Although, after the LLLT treatment, both cellular infiltration and mast cell activation were significantly reduced (Panels E and F).

The airway inflammation induced by FA inhalation caused an intense cellular infiltration in the perivascular and peribrochiolar and mast cell activation in the lung parenchyma and pleura (Fig 1C and 1D), whereas in the basal group, only resident leukocytes were observed and the mast cells were intact (Fig 1A and 1B). After the treatment with LLLT, both cellular infiltration and mast cell activation were significantly reduced (Fig 1E) (Fig 6 and Table 1).

**Table 1:** Effect of treatment with LLLT on leukocytes infiltration and mast cell activation after FA exposure.

Groups	Perivascular Infiltration (score 0–4)	Peribronquiolar Infiltration (score 0–4)	Mast cell activation (lung) (score 0–4)	Mast cell activation (pleura) (score 0–4)	Total (score 0–16)
Basal	0,6 ± 0,2	0,8 ± 0,2	0,4 ± 0,2	0,4 ± 0,2	2,4 ± 0,5
FA	3,8 ±0,2*	2,6 ± 0,2 *	2,6 ± 0,2*	3,2 ± 0,2*	12,2 ±0,4*
FA+L	1,8 ± 0,2 #	1,8 ± 0,2	1,6 ± 0,2 #	1,8 ± 0,2 #	7,0 ± 0,6 #

Group of rats was exposed or not to FA inhalation (1%, 90 min/day, 3 days) and treated or not with LLLT (3J/cm^2^, 30mW, 60s/point, total 540s, 1 and 5h post each FA inhalation). After 24 h the lungs were removed and the morphological analyses were performed. Data of score: 0 absence; 1 weak presence; 2 Average presence; 3 intense presence; and 4 very intense presence. Data are expressed as mean ± SD.

*P< 0.05 in relation to basal group

#P < 0.05 in relation FA group

## Discussion

Experimental studies with animals [4,5,17] as well as in humans [1,2,3] have reported the anti-inflammatory effects of LLLT in different lung diseases, including asthma and chronic obstructive pulmonary diseases.

The skin barrier has been always a major obstacle when applying physical energy in different human and rodent tissues [18,19]. 20% to 58% of energy penetrates into a rat’s skin surface reaching into the deep tissue area based on a photobiomodulation study [19]. Our previous works regarding the airways have been applying different wavelengths in order to treat lung and trachea disorders. We have researched both animal and human respiratory disorders looking to accomplish substantial effects when applying an adequate amount of energy [4,5,17,20,21]. In these studies, we have evaluated the local effects of LLLT. However, according to Tomimura et al [22], a systemic treatment with LLLT modulated both the inflammatory process and the oxidative stress against systemic arterial hypertension. Low level laser therapy was able to avoid endothelial dysfunction and increase nitric oxide (NO) production. Thus, both of the treatments (local and systemic) were responsible for modulating the inflammation and bringing the system into a homeostasis state.

The effects of LLLT on injured tissues, including proliferation, collagen synthesis, and the release of growth factors from cells, are caused by the energy delivered through light irradiation. However, the mechanisms of LLLT need to be greatly explored, primarily in lung diseases. Here, we have shown that treatment with LLLT reduced the lung inflammation induced by an FA exposure. The parameters of laser utilized in the present study were based on the effects of laser in different models of lung disease which showed an efficient reduction in the lung’s cellular recruitment, its cytokine secretion, mucus secretion, and collagen deposition [7,17,23]**.** Our data has therefore suggested that residual energy was enough to reach the dose threshold for anti-inflammatory action, together with the tissue repairing process, as well as stimulating the physiological state and bringing tissue into a homeostasis state.

We used an important and interesting model of lung inflammation triggered by exposure to a pollutant. Formaldehyde (FA) is an outdoor and indoor pollutant and its vulnerability can be related to environmental sources, such as building materials, combustion processes, tobacco smoke, and in occupational settings, including textiles, paint, resins, and the plastic industries. Endogenous sources can also be considered since FA is a metabolite resulting from the metabolism of amino acids and xenobiotics [13]. Thus, FA exposure constitutes a relevant model of lung inflammation since many people are exposed to this kind of pollutant.

Considering that the effects of FA in the airways are well established and cause neutrophilic lung inflammation [11,15], we investigated the anti-inflammatory effects of a treatment with LLLT in order to better understand the mechanisms involved. Our results have shown that a treatment with LLLT reduced the neutrophilic lung inflammation induced by an FA inhalation. We observed a decrement in cell influxes in the alveolar space, as well as in MPO activity, which is an index of the presence of neutrophils. Analyzing the cell mobilization processes is very complex and multi-mediated. Our results have shown that this traffic of cells from the bone marrow into the blood and into the injured tissue occur normally in animals submitted to an FA inhalation. This connection was not observed in animals treated with an LLLT, as a lower number of leukocytes were found in the blood and in the lung. However, no differences were observed in the number of cells of bone marrow. Thus, we must consider that the production and the release of leukocytes in normal bone marrow is a complex process mediated by transcription factors, cytokines such as IL-17 and IL-23, a growth-colony stimulating factor (G-CSF), chemokine receptors (CXR4 and CXCR2), and others [24,25,26]. Moreover, it is reasonable to consider that alterations in the number of cells in the bone marrow could occur before the analyses were performed (24 h after the last FA inhalation).

FA exposure induces the conformational changes in the lung microvasculature culminating in an increased lung permeability and edema [15]. Such alterations in the lung microenvironment are very harmful and contribute to the elevation of the inflammatory process. LLLT significantly reduced the increased vascular permeability in the parenchyma and in the intrapulmonary bronchi after an FA exposure.

In order to explain the pathway involved in the effect of LLLT in the lung, we evaluated the inflammatory and anti-inflammatory cytokines which modulate lung cell influxes and vascular permeability after an FA exposure. In previous studies, we have shown that FA induces an increased lung permeability modulated by leukotriene B4, thromboxane B2, interleukin 6, 1 beta, and a factor growth of endothelial vascularity [15].

Cytokines such as TNF-alpha, IL-6, and IL-10, modulate the recruitment and the activation of leukocytes in lung diseases. They also exert an important effect in lung inflammation after an FA exposure. TNF-alpha is produced by epithelium, mast cells, macrophages, and induces the migration and the activation of leukocytes and airway hyperreactivity. IL-6 plays a complex role in the inflammatory response and is associated with chronic inflammation. IL-10 is an anti-inflammatory cytokine and plays an important role in the resolution process of inflammation.

To explain the reduced inflammation and the lung permeability, the levels of pro-inflammatory cytokines in the BAL (IL-6 and TNF-alpha) were reduced after an LLLT treatment. Nevertheless, elevated concentrations of IL-10 were observed, suggesting that the profile of activated cells in the BAL was different when LLLT was used. In addition, we must consider that the reduced levels of IL-6 and TNF-alpha might to be a reflection of the reduced number of neutrophils, since such cytokines that are released by neutrophils are responsible for the amplification of the inflammatory response [27].

An elevated gene expression of IL-10 in the lung tissue was observed after the treatment with LLLT. This result is important and shows that the effects of LLLT during an FA-induced lung inflammation involved a pre-transcriptional pathway, since the IL-10 gene expressions were increased.

One of the important mechanisms which FA induces is a neutrophilic lung inflammation by mast cell degranulation [11, 28,29,30]. Mast cells are activated by FA via neuropeptides [11]. These cells constitute an immunological sentinel and once activated, they release a wide spectrum of inflammatory mediators [31]. When considering the reduced effect of an LLLT treatment in neutrophilic lung inflammation after an FA exposure, we evaluated the effect of this treatment in the mast cell degranulation. Our data has shown that an LLLT treatment significantly reduced the mast cell degranulation and this effect seems to be the major cause for the reduced lung inflammation and the reduced lung permeability during an FA inflammation.

The effect of LLLT on mast cell degranulation is controversial. Studies *in vitro* have shown that transient receptor potential vanilloids (TRPV) in mast cells are a target for LLLT culminating in an increased release of histamine [32,33]. On the other hand, Otronosova [34] showed that LLLT reduced histamines in the peripheral blood and this effect was linked with an improved external respiratory function.

In conclusion, our results have shown that LLLT reduces neutrophilic lung inflammation that has been FA-induced by a decrement of inflammatory cytokines and mast cell degranulation. This study may provide important information about the mechanisms of LLLT in lung inflammation induced by a pollutant.
